# Neuropathy-specific alterations in a Mexican population of diabetic patients

**DOI:** 10.1186/s12883-017-0939-6

**Published:** 2017-08-25

**Authors:** Angélica Carbajal-Ramírez, Rebeca García-Macedo, Carlos Manlio Díaz-García, Carmen Sanchez-Soto, Araceli Méndez Padrón, Jorge Escobedo de la Peña, Miguel Cruz, Marcia Hiriart

**Affiliations:** 1 Neurology Service “Dr. Bernardo Sepúlveda G”. Centro Médico Nacional Siglo XXI, Mexican Institute of Social Security, Mexico City, Mexico; 2 Medical Research Unit in Biochemistry, UMAE Bernardo Sepúlveda, Centro Médico Nacional Siglo XXI, Mexican Institute of Social Security, Mexico City, Mexico; 3 Department of Cognitive Neuroscience, Instituto de Fisiología Celular, Universidad Nacional Autónoma de México/Circuito Ext. SN, UNAM, CP 04510 Mexico, México City México; 4 Research Unit of Clinical Epidemiology Gabriel Mancera, Hospital Regional 1 Carlos MacGregor Sánchez Navarro, Mexican Institute of Social Security, Mexico City, Mexico

**Keywords:** Cardiovascular complications, Cell adhesion molecules, Diabetic complications, Dyslipidemia, Inflammation, Insulin resistance, Neuropathy, Neurotrophin, Renal dysfunction, Type 2 diabetes mellitus

## Abstract

**Background:**

Neuropathy is one of the major complications of type 2 diabetes mellitus. Our first aim was to determine the clinical characteristics of a population of diabetic patients with different types of neuropathy. Our next goal was to characterize the cytokine profile (IL-6 and IL-10), nerve growth factor (NGF) and circulating cell-adhesion molecules in these patients. Finally, we aimed to compare the renal function among the groups of neuropathic patients.

**Methods:**

In a cross-sectional study, we included 217 diabetic patients classified in three groups: sensory polyneuropathy with hypoesthesia (DS_h_P) or hyperesthesia (DS_H_P), and motor neuropathy (DMN). Two control groups were included: one of 26 diabetic non-neuropathic patients (DNN), and the other of 375 non-diabetic (ND) healthy subjects. The participants were attending to the Mexican Institute of Social Security.

**Results:**

The circulating levels of NGF were significantly lower in diabetic patients, compared to healthy subjects. The range of IL-6 and IL-10 levels in neuropathic patients was higher than the control groups; however, several samples yielded null measurements. Neuropathic patients also showed increased circulating levels of the adhesion molecules ICAM, VCAM, and E-Selectin, compared to the ND group. Moreover, neuropathic patients showed reduced glomerular filtration rates compared to healthy subjects (82–103 ml/min per 1.73 m^2^, data as range from 25th–75th percentiles), especially in the group with DMN (45–76 ml/min per 1.73 m^2^).

**Conclusions:**

Some particular alterations in neuropathic patients included -but were not limited to- changes in circulating NGF, cell adhesion molecules, inflammation, and the worsening of the renal function. This study supports the need for further clinical surveillance and interventions considering a neuropathy-related basis.

**Electronic supplementary material:**

The online version of this article (doi:10.1186/s12883-017-0939-6) contains supplementary material, which is available to authorized users.

## Background

Diabetes has reached epidemic numbers worldwide. The prevalence in the Mexican population is around 14%, with neuropathy as a major complication [1]. Peripheral neuropathies may result in a variety of sensory and autonomic injuries that usually need of glycemic control and pain management [2]. Furthermore, other conditions and complications are likely to be developed by diabetic patients, such as hypertension, retinopathy, and nephropathy [3].

Changes in the circulating levels of several signaling molecules have been associated with metabolic diseases. A high profile of inflammatory cytokines is involved in the physiopathology of metabolic syndrome and type 2 diabetes mellitus (T2DM). Moreover, nerve growth factor (NGF) is often altered in these pathologies and also participates in nerve fiber survival [4, 5].

T2DM is a multifactorial disease that evolves with the worsening of a plethora of functions, ranging from metabolic disorders to inflammation, nerve degeneration or compromised renal function as one of its more severe co-morbidities [3]. In spite of the huge concern about neuropathies and joint deterioration of sensory and mental functions [6], there are few comparative reports in patients with different types of diabetic neuropathy. Here, instead of focusing only on one type of neuropathy or mixing several neuropathies in a single test group, we attempted to characterize the prevalence and extent of these alterations in different types of diabetic neuropathy.

In the present study, we pursued three aims: (1) to determine the clinical characteristics of a population of diabetic patients with different types of neuropathy, (2) to characterize the levels of interleukin 6 (IL-6) and interleukin 10 (IL-10), nerve growth factor and circulating cell-adhesion molecules, and (3) to compare the renal function among the groups of neuropathic patients. We also examined healthy subjects and patients with T2DM, with or without neuropathy, according to several measurements of metabolic, hemostatic, cardiovascular, inflammatory and renal functions.

## Methods

### Study population

This study comprised 375 non-diabetic control subjects and 243 T2DM patients, who were attending to the Family Medicine Clinics from the Mexican Institute of Social Security (IMSS).

### Conduct of the study

The cross-sectional study was conducted from 2009 to 2010 in Mexico City. Among those excluded were pregnant women, patients with type-1 diabetes mellitus, kidney or heart diseases, hepatitis, peripheral arterial insufficiency diagnostic, positive HIV serology, and self-reported presence of acute infections or chronic illness such as allergies and autoimmune diseases, which could alter the circulating levels of cytokines. Also, those subjects participating in a weight reduction program were excluded. A similar population of age- and gender-matched non-diabetic, healthy subjects served as control.

We recruited patients attending the Medical Biochemistry Research Unit and outpatients attending the Neurology Service at the Hospital of Specialties of The National Medical Centre Siglo XXI. The patients were referred from family medicine clinics (numbers 5, 7, 9, 15, 16, 21, 22, 26, 38, 79 and 161), corresponding to the 03 delegation of IMSS, with a diagnosis of T2DM according to the criteria of the American Diabetes Association (ADA) or World Health Organization consultation criteria [7, 8].

### Classification criteria for diabetic neuropathies

**Fig. 1:**
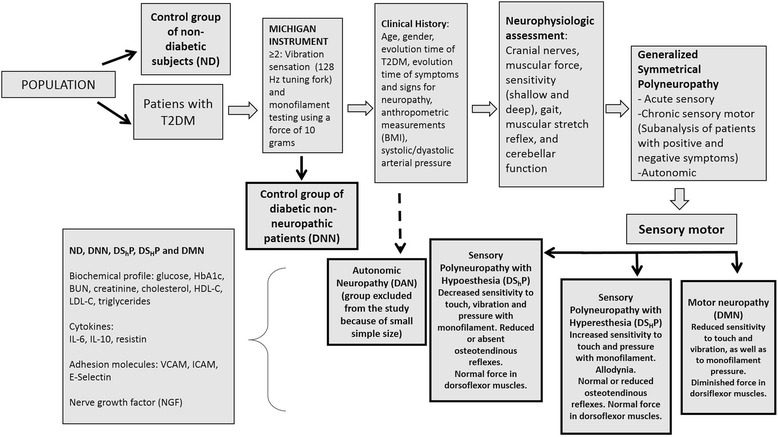
Flowchart of the classification criteria for diabetic neuropathies. Patients were screened for diabetic neuropathy using the Michigan Neuropathy Screening Instrument (MNSI) according to the criteria of the consensus of San Antonio

A total group of 649 patients from the IMSS Family Medicine Clinics, fulfilling ADA criteria for T2DM, were screened for neuropathy, using the Michigan Neuropathy Screening Instrument (MNSI) [9], with high specificity (88.4%) and sensitivity (78.1%) according to the criteria of the consensus of San Antonio [9]. Patients with a screening value ≥2 were considered neuropathic [10], as summarized in Fig. 1. Risk factors evaluated by the Michigan Neuropathy Screening Instrument (MNSI) were: age, time of evolution of diabetes, high-density lipoproteins-cholesterol (HDL-Cholesterol) and glycosylated hemoglobin (HbA1c). MNSI can be used as a relatively simple and reliable method for clinical and epidemiological screening and assessment of asymptomatic diabetic peripheral neuropathy (ADPN), as previously reported [7], with high specificity albeit lower sensitivity compared to nerve conduction velocity tests [8].

Furthermore, patients with diabetic neuropathy were submitted to a complete neurological review to determine sensory or motor neuropathy. Sensory neuropathy was characterized by the presence of feeling of numbness, burning pain, lack of feeling in the feet, cramps, feeling of itching, pain to cover their feet with the sheet, the ability to discriminate the temperature level at their feet during bathing, the presence of wounds or ulcers, exacerbation of discomfort during the afternoon to night, their ability to feel their feet when walking, the presence of dryness or cracks on their feet or amputation, the reduction or loss of sensation to touch and/or temperature (which is symmetrical in glove and sock), unique loss of thermal sensitivity (even though this variety is very rare), reduction or loss of the vibration sense, as well as the sense of position, usually by affecting the myelinated long fibers and other proprioceptive afferent fibers.

The presence of alterations in small fibers is associated with increase in the sensation of temperature, the presence of pain, a feeling of painful burn and some symptoms related to significant autonomic alteration due to damage of unmyelinated fibers. Motor neuropathy was characterized by the presence of weakness in the extremities (usually distal), accompanied or non-by altered sensibility [11].

The group of patients with sensory neuropathy was sub-classified in a group with hypoesthesia or less sensitivity, and hyperesthesia with greater sensitivity, to identify differences between these groups. Patients with sensory polyneuropathy and hypoesthesia were characterized by decreased sensitivity to touch, vibration and pressure with monofilament; as well as reduced or absent osteotendinous reflexes. By contrast, patients with sensory polyneuropathy and hyperesthesia were characterized by increased sensitivity to touch, vibration and pressure with monofilament; as well as normal or reduced osteotendinous reflexes. A landmark for this group was the manifestation of allodynia. The neuro-conduction studies were not taken into consideration because we did not identify any atypical clinical picture, such as superimposed chronic inflammatory demyelinating polyneuropathy (CIDP), and there was no case of any familial history of neuropathy [12].

Diagnosis of cardiovascular autonomic neuropathy was documented by previous cardiology review, with tachycardia at rest, physical exercise intolerance, orthostatic hypotension and silent myocardial ischemia. Patients answered a questionnaire to evaluate alteration in esophageal motility, diagnosis of gastroparesis diabeticorum, constipation, diarrhea or fecal incontinence, and genitourinary disorders like diabetic cystopathy, erectile dysfunction, and retrograde ejaculation, also, sexual dysfunction with loss or reduction of vaginal lubrication [13]. Patients showed autonomic neuropathy if they presented the following symptoms: chronic diarrhea (predominantly at night), gustatory salivation, dizziness on rising, erectile dysfunction and orthostatic hypotension by reducing 30 or more mmHg in systolic pressure in the morning [14, 15].

The patients also answered a questionnaire with 15 questions (options yes or not). Those who answered positively for the presence of resting tachycardia, intermittent blurry vision, posture instability, syncope, postprandial hypotension and intolerance to physical exercise, were further analyzed for their electrocardiograms, deep breathing, heart rate variability and orthostatic hypotension [9, 16–18]. Only seven patients were diagnosed with pure autonomic neuropathy (DAN) in the original group. We excluded this neuropathic group from the study because of the scarce sample size.

A complete medical history and neurological examination included cranial nerves, sensory and motor functions, as well as tendon reflexes. The vibration sense was evaluated with a tuning fork of 128 Hz, and a filament of 10 g tensiometer was used to evaluate the light touch perception. Polyneuropathy was defined as a failure to elicit the knee or Achilles muscle stretch reflexes with or without symptoms of sensory disturbance on both feet; or typical loss of sensation to pin prick and distal deep pain and/or loss of proprioception with sensory ataxia and a positive Romberg sign and/or burning sensation in the feet at night or day [14, 19].

Patients were classified after a complete physical-neurological examination, in the following groups: A control group of non-diabetic subjects (ND), a control group of diabetic non-neuropathic patients (DNN) and three test groups that included patients with sensory polyneuropathy with hypoesthesia (DS_h_P) or hyperesthesia (DS_H_P), as well as patients with motor neuropathy (DMN). As the total number of measurements could not be eventually assessed in all the individuals, a random selection within each group was performed.

### Clinical measurements

Anthropometric indexes: Body weight was taken using a digital scale (Seca, Hamburg, Germany) to the nearest 0.1 kg and height was measured using a Seca 255 stadiometer to the nearest 0.1 cm. Body mass index was calculated as previously reported [20].

Blood pressure measurements: Systolic and diastolic blood pressures were obtained by auscultation using a sphygmomanometer (ALPK2, Tokyo, Japan) with appropriate cuff size for arm length. Four blood pressure readings were taken for each participant in the right arm in sitting position, resting 1 min between measurements, and considering the final levels of systolic and diastolic blood pressures (SP and DP, respectively) as the mean of the last three readings for each case. The standard equation was used to calculate the mean arterial pressure (MAP), as follows: MAP = DP+(SP-DP)/3 [21].

Clinical biochemistry: For biochemical analysis, blood was drawn after a 12 h fast. Glycated hemoglobin, serum glucose, total cholesterol, low-density lipoprotein cholesterol, high-density lipoprotein cholesterol and triglycerides were measured using an auto-analyzer instrument (Instrumentation Laboratory, ILAB 350, Barcelona, Spain). Plasma interleukin-6, interleukin-10, resistin, intercellular adhesion molecule-1, vascular adhesion molecule-1 and E-selectin were determined by standard enzyme-linked immunosorbent assay (ELISA) kits with inter-assay coefficients of variation <9% (R & D Systems, NE, Minneapolis, USA).

Creatinine and blood urea nitrogen were also assessed, and the Glomerular Filtration Rate (GFR) was calculated using the quadratic GFR equation as described elsewhere [22].

NGF determination: NGF concentration in plasma was measured by ELISA using the DuoSet kit (R & D Systems, NE, Minneapolis, USA), according to manufacturer instructions.

### Statistical analysis

Zero values in IL-6 and IL-10 measurements were numerous in several groups of diabetic patients. The percentages of zero values were analyzed independently of non-zero values, as a simple approach to cope with data containing many null measurements [23].

Data management and analysis were performed double blind. Frequency distributions were analyzed using a Chi-squared test with Yate’s correction for continuity on 2 × 2 contingency tables. Working data were tested for normality by a Kolmogorov-Smirnov test. Most of the measurements did not fulfill normality, even after logarithmic or square root transformations for correcting normality and variance heterogeneity [24]. Comparisons among groups were performed by the non-parametric Kruskal-Wallis test (with a Dunn post test), using the statistical software GraphPad InStat version 3.00 (San Diego California USA). Datasets used for analysis are provided as a Additional file 1 (Table S1).

Different letters indicate statistical differences among groups (i.e. in five groups denoted as “a”, “b”, “c”, “ab” and “abc”, the groups “a”, “b”, “c” are statistically different from each other, the group “ab” only differs from “c” and the group “abc” is not significantly distinct from any other). Sample sizes may differ among conditions within the same group due to unavailability of individual blood sample for all the measurements. Graphics were constructed using Origin version 8 (Northampton USA).

## Results

### Clinical characteristics of study participants

Demographic and clinical characteristics of the study groups are summarized in Table 1. The average age was similar, except for DMN and DNN patients, with the highest and lowest values, respectively. Female gender proportion was more than a half in each group, with the highest percentage in the ND group (75%). According to a Chi-squared test, the percentages of female gender from the DS_h_P (63%) and the DMN (56%) groups significantly differ from ND subjects, but there were not differences among the diabetic groups.

**Table 1:** Demographic and Clinical Characteristics of Non-Diabetic controls (ND) and Diabetic Non-Neuropathic Subjects (DNN), as well as Patients with Sensory Polyneuropathy with Hypoesthesia (DS_h_P) or Hyperesthesia (DS_H_P), and Diabetic Motor Neuropathy (DMN)

	ND	DNN	DS_h_P	DS_H_P	DMN
Demographic characteristics	*N* = 375	*N* = 26	*N* = 121	*N* = 55	*N* = 41
Age (years)	56 (49–63) ^bc^	50 (42–59) ^c^	58 (52–63) ^ab^	56 (52–61) ^abc^	61 (55–65) ^a^
Female gender (%)	75 (281/375) ^a^	69 (18/26) ^ab^	63 (76/121) ^b^	62 (34/55) ^ab^	56 (23/41) ^b^
Medical history					
Duration of Diabetes (years)	-	4 (2–8) ^b^	8 (3–13) ^ab^	10 (4–15) ^a^	13 (5–18) ^a^
Hypertension (%)	-	12 (3/26) ^a^	21 (25/121) ^a^	24 (13/55) ^a^	27 (11/41) ^a^
Medical treatment					
Oral Hypoglycemic Drugs (%)	-	96 (25/26) ^ab^	93 (112/121) ^a^	89 (49/55) ^ab^	78 (32/41) ^b^
Insulin treatment (%)	-	8 (2/26) ^b^	17 (21/121) ^b^	27 (15/55) ^ab^	34 (14/41) ^a^
PPAR-α/γ Activators pioglitazone or bezafibrate (%)	-	42 (11/26) ^a^	20 (24/121) ^b^	16 (9/55) ^b^	22 (9/41) ^ab^
HMGCoA reductase Inhibitors (%)	-	23 (6/26) ^a^	12 (15/121) ^a^	11 (6/55) ^a^	15 (6/41) ^a^

Different letters indicate statistical differences among groups with a *p* < 0.05, using a Kruskal-Wallis test with a Dunn post test. Data expressed as percentages were analyzed using a Chi-squared test with Yate’s correction for continuity on 2 × 2 contingency tables. Data are represented as median (25th – 75th percentiles of the distribution), except for percentages, which are represented as percentage (number of patients fulfilling the condition/sample size, N)

Other differences were found after physical examinations (Table 2); i.e., the BMI was most elevated in diabetic non-neuropathic patients and the group with sensory polyneuropathy with hypoesthesia, compared to healthy subjects. Although the percentages of previously diagnosed hypertensive patients did not differ between the groups of diabetic patients, the mean arterial pressures were significantly elevated in the neuropathic groups respect to ND subjects.

**Table 2:** Physical examination and metabolic findings

	ND	DNN	DS_h_P	DS_H_P	DMN
Body Mass Index (kg/m^2^)	27 (25–29) ^b^	30 (28–33) ^a^	28 (26–33) ^a^	29 (26–33) ^ab^	27 (25–30) ^ab^
	(*N* = 267)	(*N* = 17)	(*N* = 87)	(*N* = 39)	(*N* = 38)
Mean Arterial Pressure (mm Hg)	90.0 (83.3–93.3) ^b^	90.0 (83.3–96.7) ^ab^	93.3 (86.7–103.3) ^a^	96.7 (91.7–103.3) ^a^	95.0 (86.7–103.3) ^a^
	(*N* = 165)	(*N* = 17)	(*N* = 87)	(*N* = 39)	(*N* = 38)
Glucose (mg/dl)	86 (77–97) ^b^	124 (104–151) ^a^	130 (100–172) ^a^	143 (112–182) ^a^	136 (113–192) ^a^
	(*N* = 363)	(*N* = 20)	(*N* = 109)	(*N* = 50)	(*N* = 40)
HbA1c (%)	5.42 (4.96–5.99) ^b^	6.59 (6.26–7.88) ^a^	7.39 (6.06–9.41) ^a^	8.84 (6.82–10.62) ^a^	8.58 (6.89–10.18) ^a^
	(*N* = 360)	(*N* = 20)	(*N* = 108)	(*N* = 50)	(*N* = 39)

Different letters indicate statistical differences among groups with a *p* < 0.05, using a Kruskal-Wallis test with a Dunn post test. Data are represented as median (25th – 75th percentiles of the distribution), and the sample size (N) is shown in the row below. Sample sizes may differ among rows within the same column (group) due to unavailability of individual blood sample for all the measurements

### Glucose homeostasis and dyslipidemia in neuropathic patients

Fasting glucose levels were significantly increased in all groups of diabetic patients compared to healthy subjects (Table 2). Glycated hemoglobin (HbA1c), expressed as the percentage of total hemoglobin, was analyzed as an index of long-term insulin resistance (Table 2). Values of this parameter for patients with DS_H_P and DMN were again higher than the rest of the groups, followed in decreasing order by DS_h_P, DNN and ND. Hypertriglyceridemia was also observed in all the diabetic groups (Table 3). In addition, while the median total cholesterol was below 200 mg/dl in all groups, high-density lipoprotein (HDL-Cholesterol) levels were reduced in patients presenting sensory polyneuropathy with hypoesthesia. Even though the low-density lipoprotein levels (LDL-Cholesterol) were similar among groups, the DS_H_P group presented the lowest average in the HDL/LDL ratio, followed by patients with DS_h_N (Table 3).

**Table 3:** Lipid profile

	ND	DNN	DS_h_P	DS_H_P	DMN
Triglycerides (mg/dl)	116 (86–159) ^b^	166 (106–203) ^ab^	144 (123–198) ^a^	153 (116–219) ^a^	171 (129–232) ^a^
	(*N* = 363)	(*N* = 20)	(*N* = 109)	(*N* = 50)	(*N* = 40)
Cholesterol (mg/dl)	180 (149–207) ^a^	153 (132–201) ^a^	167 (147–198) ^a^	190 (156–213) ^a^	172 (162–214) ^a^
	(*N* = 363)	(*N* = 20)	(*N* = 109)	(*N* = 50)	(*N* = 40)
HDL-Cholesterol (mg/dl)	46 (38–57) ^a^	42 (38–50) ^ab^	42 (35–50) ^b^	43 (34–53) ^ab^	47 (38–57) ^ab^
	(*N* = 361)	(*N* = 19)	(*N* = 108)	(*N* = 50)	(*N* = 40)
LDL-Cholesterol (mg/dl)	119 (98–140) ^a^	113 (87–148) ^a^	114 (96–138) ^a^	130 (107–159) ^a^	112 (95–132) ^a^
	(*N* = 359)	(*N* = 19)	(*N* = 107)	(*N* = 50)	(*N* = 40)
HDL / LDL	0.39 (0.31–0.51) ^a^	0.39 (0.28–0.53) ^ab^	0.35 (0.30–0.45) ^ab^	0.32 (0.27–0.39) ^b^	0.37 (0.30–0.50) ^ab^
	(*N* = 359)	(*N* = 19)	(*N* = 107)	(*N* = 50)	(*N* = 40)

Different letters indicate statistical differences among groups with a *p* < 0.05, using a Kruskal-Wallis test with a Dunn post test. Data are represented as median (25th – 75th percentiles of the distribution), and the sample size (N) is shown in the row below. Sample sizes may differ among rows within the same column (group) due to unavailability of individual blood sample for all the measurements

### Hormonal and inflammatory signals are altered in diabetic neuropathies

**Fig. 2:**
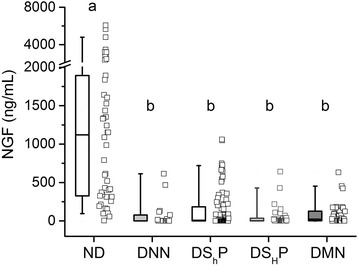
Circulating levels of nerve growth factor. Nerve growth factor (NGF) is diminished in diabetic patients respect to non-diabetic subjects. Data are represented as box plots with the median and the 25th–75th percentiles of the distribution. Whiskers comprise the 5th–95th percentiles of the distribution. The raw data is plotted to the right of box plots for all conditions: ND (*N* = 46), DNN (*N* = 18), DS_h_P (*N* = 84), DS_H_P (*N* = 38) and DMN (*N* = 38)

We further explored some hormones and cytokines, to determine if their levels vary according to the type of neuropathy. The median circulating NGF level was reduced in more than 90% in all the groups of diabetic patients, compared to healthy controls. Among them, the DS_H_P group exhibited the lowest values (Fig. 2).

**Fig. 3:**
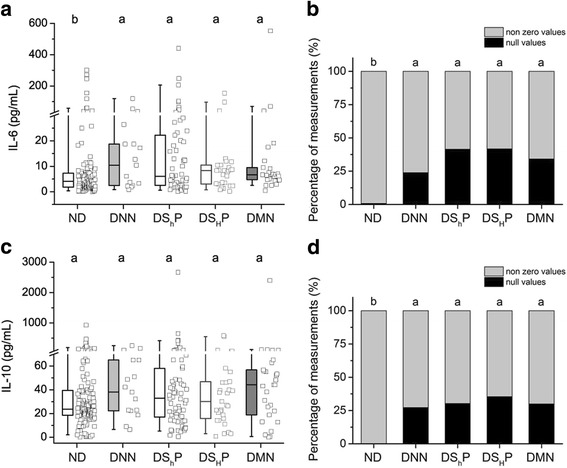
Circulating levels of cytokines. (**a**) Box-plots of pro-inflammatory cytokine IL-6 levels (for non-zero values) showed considerable variability in all groups, although a significant increase was observed in diabetic patients. Data are represented as the median and the 25th–75th percentiles of the distribution. Whiskers comprise the 5th -95th percentiles of the distribution. The raw data is plotted to the right of box plots for all conditions: ND (*N* = 127), DNN (*N* = 16), DS_h_P (*N* = 58), DS_H_P (*N* = 28) and DMN (*N* = 27). Different letters indicate statistical differences with a *p* < 0.05 and the alphabetical order points toward a decrease in magnitude (i.e. the “a” correspond to the highest value, the “b” to the second, and so on). Groups were compared by a Kruskal-Wallis non-parametric test, using a Mann-Whitney post test for a higher the statistical power with respect to Dunn test (**b**) A high percentage of measurements of IL-6 in in diabetic patients yielded null values. (**c**) Box-plots of IL-10 levels (non-zero values) were similar among groups; ND (*N* = 146), DNN (*N* = 16), DS_h_P (*N* = 69), DS_H_P (*N* = 31) and DMN (*N* = 28). (**d**) A high percentage of measurements of IL-10 in diabetic patients also yielded null values. The percentages were analyzed using a Chi-squared test with Yate’s correction for continuity on 2 × 2 contingency tables

The distribution of IL-6 levels exhibited a wide range of values in diabetic patients; however, the high proportion of null values in these groups hindered the statistical analysis. To approach this problem most directly, we analyzed zero and non-zero values as recommended elsewhere [23]. Indeed, the ranges of non-zero measurements in diabetic patients were significantly higher than in non-diabetic subjects (Fig. 3a). Indeed, the high percentages of null IL-6 values in diabetic patients (24% in DNN, 41% in DS_h_P, 42% in DS_H_P and 34% in DMN), contrasted with a very low percentage of zero values (0.8%) in the group of ND subjects (Fig. 3b).

A similar scenario occurred for IL-10, where most of the groups exhibited high percentages of zero measurements (27% in DNN, 30% in DS_h_P, 35% in DS_H_P and 30% in DMN), contrary to the ND group with no null measurements (Fig. 3d). The distributions of non-zero measurements were similar among all groups (Fig. 3c).

### Cell-adhesion molecules levels vary according to the type of diabetic neuropathy

Some cell-adhesion proteins in plasma have been described as predictors of diabetic neuropathy [25]. Interestingly, all the groups of diabetic patients show increased levels in the vascular adhesion molecule-1 (VCAM) compared to healthy subjects (Table 4), where the DMN group displayed the highest value. The inter-cellular adhesion molecule-1 (ICAM) was also elevated in all diabetic groups in >70%, compared to the ND group, although no differences were found among the non-neuropathic patients and those with diabetic neuropathies (Table 4). Median levels of E-selectin also increased between 131 and 311% in diabetic patients, compared to healthy subjects (Table 4). Again, patients with DMN and DS_H_N presented the highest values.

**Table 4:** Cell adhesion molecules are increased in diabetic neuropathy

	ND	DNN	DS_h_P	DS_H_P	DMN
Vascular adhesion molecule-1 (ng/ml)	127 (83–263) ^a^	296 (272–327) ^bc^	293 (259–335) ^c^	312 (284–367) ^bc^	353 (298–389) ^b^
	(*N* = 160)	(*N* = 21)	(*N* = 98)	(*N* = 49)	(*N* = 40)
Inter-cellular adhesion molecule-1 (ng/ml)	103 (60–143) ^b^	185 (158–227) ^a^	178 (150–213) ^c^	191 (167–240) ^a^	194 (156–243) ^a^
	(*N* = 159)	(*N* = 21)	(*N* = 98)	(*N* = 49)	(*N* = 40)
E-Selectin (ng/ml)	17 (9–23) ^b^	38 (19–60) ^a^	46 (29–68) ^a^	60 (32–105) ^a^	68 (39–97) ^a^
	(*N* = 160)	(*N* = 21)	(*N* = 98)	(*N* = 49)	(*N* = 40)

Different letters indicate statistical differences among groups with a *p* < 0.05, using a Kruskal-Wallis test with a Dunn post test. Data are represented as median (25th – 75th percentiles of the distribution), and the sample size (N) is shown in the row below. Sample sizes may differ among rows within the same column (group) due to unavailability of individual blood sample for all the measurements

### Patients with diabetic neuropathy exhibit an altered kidney function

**Fig. 4:**
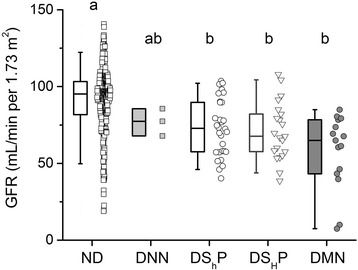
Renal function is impaired in diabetic neuropathy. (a) Glomerular Filtration Rate is decreased in all the groups of neuropathic patients. Data are represented as box plots showing the median and the 25th–75th percentiles of the distribution. Whiskers comprise the 5th–95th percentiles of the distribution. The raw data is plotted to the right of box plots for all conditions: ND (*N* = 361), DNN (*N* = 3), DS_h_P (*N* = 30), DS_H_P (*N* = 21) and DMN (*N* = 15). Different letters indicate statistical differences with a *p* < 0.05 and the alphabetical order points toward a decrease in magnitude (i.e. the “a” correspond to the highest value, the “b” to the second, and so on). Groups were compared by a Kruskal-Wallis non-parametric test, using a Dunn post test

We explored kidney function, since nephropathy is a frequent complication in T2DM [3]. The glomerular filtration rate (GFR) was calculated to determine signs of renal dysfunction. The data suggest that although all the neuropathic patients show a reduced GFR, the most affected are those with motor neuropathy (Fig. 4).

## Discussion

We analyzed four groups of diabetic patients with different types of neuropathy and compared their metabolic performances with respect to healthy subjects and diabetic non-neuropathic patients. We characterized the most prominent alterations in these groups to identify potential targets for improved treatments on a case-specific basis.

We observed a severe impairment in glucose homeostasis of patients with motor neuropathy, as well as sensory polyneuropathy with hyperesthesia. Hyperglycemia causes cellular damage through advanced glycation end products or direct activation of oxidative stress [2]. The increased levels of glycated hemoglobin in diabetic patients reflect a sustained state of insulin resistance, despite the high percentages of subjects being treated with exogenous insulin in these groups.

Glycated hemoglobin altogether with hypertension and low HDL-Cholesterol levels show a direct relationship with retinopathy; and HbA1c levels above 6.6% have been also associated with other microvascular complications, such as chronic kidney disease and peripheral neuropathy [26]. This study shows that diabetic patients with median levels of glycated hemoglobin equal or greater than 7.39%, present different kinds of neuropathies. Our results are in agreement with previous reports indicating an association between neuropathy and glycated hemoglobin [27].

Even if a correct glycemic control could be achieved, the contribution of chronic neuronal insults such as dyslipidemia, hypertension, and inflammation, could account for worsening of neuropathy [2]. Higher mean arterial pressure values in the neuropathic groups suggest the presence of these complications.

Dyslipidemias have been related to pro-apoptotic and pro-inflammatory events leading to nerve injury, and eventually to neuropathy [2, 28]. As expected, triglyceride levels were increased in patients with T2DM, but without differences between groups. Patients with sensory neuropathies, especially those with hyperesthesia, showed a reduced HDL/LDL ratio. These factors may contribute to developing diabetic neuropathy by increasing the oxidative damage in neurons [29].

Alterations in NGF may also contribute to the development of diabetic neuropathy, as shown in animal models where NGF levels are reduced [5]. NGF is reduced in diabetic patients, and this is accentuated in patients with DS_H_N, suggesting a connection with this neuropathy.

Our results contrast with those obtained by Kim et al., who observed increased plasmatic NGF levels in diabetic patients with neuropathy, compared to patients without neuropathy and non-diabetic subjects [30]. However, these authors found that NGF levels decrease proportionally to the severity of diabetic neuropathy [30]. The contradiction could be a result of differences in the populations, i.e. ethnicity and metabolic status of the patients in our study, who presented lower duration of diabetes and systolic blood pressure, but a higher BMI. Moreover, Kim et al. used a control group of non-diabetic subjects that was measured in a different subset of experiments, contrary to our control group, which was conceived in the same experimental design and evaluated simultaneously, under the same criteria and conditions of the diabetic groups.

Inflammation could also be a target for the treatment of neuropathy in T2DM [31, 32]. It has been reported that IL-6 is elevated in diabetic patients with neuropathy [31]. It is elevated in patients with diabetic polyneuropathy and correlates with several neuropathic deficits such as impaired ankle reflex, pain perception, and a high threshold for vibration perception [33]. Indeed, we observed a higher range of IL-6 levels in diabetic patients. Nevertheless, we also observed a higher proportion of null-measurements in these groups. The low values in diabetic patients could arise from glucose-normalizing interventions since it has been demonstrated that insulin treatment decreases IL-6 levels in diabetic patients [34].

A more drastic -and antagonistic- interpretation could be a compromised immune function, especially in the light of high percentages of null measurements in IL-10 as well. For non-zero values, we did not observe differences between ND subjects and the diabetic groups. The relevance of circulating levels of IL-10 is somehow controversial. Some studies have proposed a protective role for IL-10 in diabetic retinopathy [35] and a mouse model of diet-induced insulin resistance [36]. On the other hand, it has also been reported an association between diabetic neuropathy and a single nucleotide polymorphism in the IL-10 gene that results in higher production of this cytokine [37].

Cell adhesion molecules are potential players in the development of diabetic neuropathy since these proteins are increased in patients with reduced peroneus nerve conduction velocity [25]. Accordingly, we observed that patients with DMN display elevated VCAM and E-Selectin levels compared to healthy subjects and other groups of diabetic patients.

In general, our evidence supports the possibility of diabetic motor neuropathy as a late and severe event in the pathogenesis of T2DM. This group showed the highest duration of diabetes and the most altered profile of the cell-adhesion molecules, in spite of a similar glycemic control and lower dyslipidemia compared to other groups. Accordingly, the GFR reached the lowest values in this group.

## Conclusions

Several articles have attempted to elucidate the alterations that contribute to diabetic neuropathy. However, very few of them separated population of patients in groups according to their type of neuropathy. We studied three types of diabetic neuropathy and included a group of non-diabetic subjects and a group of diabetic non-neuropathic patients as controls. We recommend replicating these results in other populations, as well as in prospective studies, for estimating how each variable (or a combination of them) may affect the risk to develop a particular type of neuropathy.

Among the subjects with sensory neuropathies, those with hyperesthesia exhibited a marked impairment in the glycemic control, dyslipidemia and a severely low level of NGF. In our study, diabetic patients with motor neuropathy displayed a compromised renal function, which could reflect an initial step in the development of renal failure. Our results shed light in the major neuropathy-specific alterations of diabetic patients. The latter may help to orient more effective interventions to prevent or treat these diabetic complications.

## Additional file

Additional file 1: Patients database. This is the database of patients, organized by groups of study. According to responsible clinical data sharing, the available data were de-identified. (XLSX 104 kb)

## Abbreviations

BMI: Body mass index
BUN: Blood urea nitrogen
DMN: Diabetic motor neuropathy
DNN: Diabetic non-neuropathic patients
DS_H_P: Diabetic Sensory Polyneuropathy with Hyperesthesia
DS_h_P: Diabetic Sensory Polyneuropathy with Hypoesthesia
GFR: Glomerular Filtration Rate
HbA1c: Glycated hemoglobin
HDL-Cholesterol: High-density lipoprotein
HMGCoA: 3-hydroxy-3-methyl-glutaryl-coenzyme A
ICAM: Inter-cellular adhesion molecule-1
IFCC: International Federation of Clinical Chemistry
IL-10: Interleukin-10
IL-6: Interleukin-6
LDL-Cholesterol: Low-density lipoprotein
ND: Non-diabetic subjects
NGF: Nerve growth factor
PPAR-γ: Peroxisome Proliferator-Activated Receptor-gamma
T2DM: Type 2 diabetes mellitus
TNF-α: Tumor Necrosis Factor-alpha
VCAM: Vascular adhesion molecule-1
